# Prasugrel 5 mg in acute coronary syndromes: indications and clinical outcomes

**DOI:** 10.1590/1806-9282.20260528

**Published:** 2026-09-21

**Authors:** Luiz Fernando Leite Tanajura, José Henrique Delamain, André Labrunie, Gabriel Brandão Neves de Souza, Luiz Gustavo de Oliveira Tanajura

**Affiliations:** 1 Institute Dante Pazzanese of Cardiology, Department of Invasive Cardiology – São Paulo (SP), Brazil.

Acute coronary syndromes (ACS) represent a major public health problem in the Western world. Recent North American statistics have clearly demonstrated their relevance: a myocardial infarction (MI) occurs every 40 s, totaling more than one million events per year, a third of which are recurrent. About a third of deaths worldwide are of cardiac origin, and although there is no specific accounting of ACS in these events, it seems clear to infer that a significant proportion of them stem from it. Thus, interventions that minimize these risks are of great relevance from both a clinical and economic perspective, justifying specific investigations^1–3^.

Early in the last decade, it was demonstrated that dual antiplatelet therapy administered exclusively orally was superior to the isolated prescription of acetylsalicylic acid (ASA) in the passivation of ACS. The P2Y12 receptor inhibitor initially used, clopidogrel, quickly gained the support of the main guidelines. Although endowed with proven efficacy, it was found that this drug had relevant drawbacks: (1) a slow onset of action; (2) inhibition of platelet aggregation below the desired level; and (3) inconsistent effect, especially in the presence of genetic polymorphisms that interfere with its metabolism. These restrictions led to the development of new medications aimed at overcoming them. Two new drugs were then developed: prasugrel and ticagrelor. In this review, the focus will be on prasugrel in its reduced dose of 5 mg/day^2,4^.

## PHARMACOLOGY OF PRASUGREL

It is a medication restricted to the ACS setting. It is a prodrug, meaning that the active agent is a metabolite derived from its hepatic metabolism. It is administered as a single daily dose, and its effect is irreversible. Like clopidogrel, it is administered as a single daily dose, having the advantages of acting earlier and more intensely than it^5^.

To prove these advantages in clinical practice, a large multicenter clinical trial in ACS was designed, comparing it to clopidogrel. In this study, TRITON TIMI 38, prasugrel was administered with a loading dose of 60 mg, followed by a maintenance dose of 10 mg/day; the protocol recommended that all included patients undergo an invasive strategy and subsequent percutaneous coronary intervention (PCI), being maintained on dual antiplatelet therapy for one year. At the end of this period, the composite primary endpoint of death, MI, or stroke was significantly reduced by 19% with prasugrel, especially due to the reduction of MI during follow-up (5.8 vs. 9.5%; p<0.001). Due to its greater potency, cases treated with this medication presented an excess of TIMI major bleeding (2.4 vs. 1.8%; p=0.03)^6^.

Regarding subanalyses, diabetic patients showed greater benefit compared to those without this disease. Two other relevant subanalyses involved subgroups whose results were neutral: the elderly over 75 years of age and those weighing less than 60 kg, groups that proved to be more predisposed to developing significant bleeding complications. As a result, a new 5 mg presentation of the drug was developed, specifically indicated for use in these subgroups^6,7^.

This new presentation was initially evaluated in the FEATHER pharmacodynamic clinical trial, encompassing patients with stable coronary disease analyzed by body weight below or above 60 kg. The involved patients could use the two doses of prasugrel or clopidogrel 75 mg. The 5 mg presentation (in those weighing <60 kg) demonstrated a similar capacity to the 10 mg dose (in those weighing >60 kg) in reducing platelet aggregation; both presentations were superior to clopidogrel in platelet inhibition^7^.

Subsequently, another study, GENERATIONS, involving cases with a focus on the objective measurement of platelet aggregation in different age groups, demonstrated that the 5 mg presentation has an antiplatelet action non-inferior to that of 10 mg in patients under 65 years of age and superior to that of clopidogrel in older age cases (equal to or greater than 75 years), in a cohort of 155 patients with stable coronary disease^8^.

## CLINICAL STUDIES IN ACUTE CORONARY SYNDROMES

### Trilogy acute coronary syndromes study^9^

A multicenter, randomized clinical trial designed to compare prasugrel versus clopidogrel in patients with non-ST-segment elevation ACS who were not revascularized. Of the 9,326 randomized cases, 22% were aged >75 years and 13% weighed less than 60 kg; in these individuals, randomization to prasugrel implied the prescription of the 5 mg/day dose. In the study's overall sample, at 17 months of follow-up, the use of the more contemporary medication was not accompanied by a significant clinical benefit regarding the occurrence of cardiovascular death, MI, or stroke. No impact on major bleeding was observed either. In the publication, there is no mention of superior clinical outcomes compared to clopidogrel in those who used the lower dose of prasugrel, both in the analysis of major events and bleeding. Subsequently, an editorial mentioned that the clopidogrel group had non-significant increases of 2 and 5%, respectively, in major cardiovascular events and bleeding. Thus, in patients who did not undergo revascularization, prasugrel was not superior to clopidogrel in reducing the primary endpoint^9,10^.

### Elderly acute coronary syndromes 2 study^11^

Designed to compare prasugrel 5 mg versus clopidogrel 75 mg after PCI in the setting of ACS with or without ST-segment elevation, in patients aged >74 years (mean age of 80 years in both groups). The composite primary endpoint consisted of death, MI, stroke, rehospitalization for new ACS, or bleeding; the sample size calculation planned to include 2,000 patients, with the hypothesis being the superiority of prasugrel. When 1,443 cases were included, the trial was prematurely terminated after an interim analysis due to the lack of clear clinical benefit with either treatment (prasugrel 17.0% versus clopidogrel 16.6%; p=NS). Thus, in this specific trial, prasugrel was not shown to be superior to clopidogrel.

### Host Reduce Polytech study^12^

A study that used the 5 mg dose of prasugrel as an alternative for P2Y12 inhibitor de-escalation. This strategy entails maintaining the usual dose of 10 mg/day for the first 30 days, aiming to reduce major cardiovascular events, which would be more common in this phase. After this period, the dose would be reduced to 5 mg/day, with the primary purpose of preventing bleeding complications. Thus, the 2,338 patients included in South Korean hospitals, all with ACS and revascularized via PCI, were randomized to maintain the usual 10 mg dose or the reduced dose 30 days after inclusion in the study. The primary endpoint of the study was composed of death, MI, stroke, stent thrombosis, new revascularization, or bleeding at 1 year of follow-up. The hypothesis was the non-inferiority of the de-escalation strategy. A 30% reduction in the composite endpoint was observed in the de-escalation group (p<0.0001 for non-inferiority), with no major impact on ischemic components but with a 52% reduction in major bleeding with de-escalation (p<0.0007). Thus, prasugrel de-escalation appears to be a valid option to reduce bleeding complications in ACS patients.

### Isar React 5 study^13^

This was a prospective, multicenter, randomized, investigator-initiated clinical trial with the objective of comparing prasugrel versus ticagrelor in the ACS setting. Due to differences in the number of daily doses between the evaluated drugs, it was not double-blind. ACS cases with or without ST-segment elevation for which an invasive strategy was indicated were included. Those with active bleeding, need for chronic use of oral anticoagulants, history of stroke or transient ischemic attack, on dialysis, and cases presenting significant hepatic dysfunction were excluded. Prasugrel was prescribed with the usual loading dose of 60 mg, followed by a maintenance dose of 10 mg/day; those aged over 75 years and/or weighing less than 60 kg were maintained on the 5 mg/day presentation. Ticagrelor was used with a loading dose of 180 mg, with a maintenance dose of 90 mg every 12 h. Both were to be maintained for one year. As determined by the Guidelines, in cases with ST-segment elevation, the loading dose of both medications was to be administered as soon as randomization occurred; in non-ST-elevation situations, ticagrelor could be administered immediately, while prasugrel could only be used after diagnostic coronary angiography. The composite primary endpoint was the combination of death, MI, or stroke at one year of follow-up, which, contrary to the hypothesis formulated by the investigators, showed a significant reduction with prasugrel. When the individual results of the different components were broken down, a significant reduction in MI cases was observed in this group, while the other two items (mortality and stroke) did not differ significantly. Major bleeding also did not differ. A pre-specified subanalysis involving only those aged >75 years or weighing <60 kg (1,099 cases, 27.3% of the study sample) discriminated the results of the 5 mg presentation against the usual dose of ticagrelor. The endpoint aggregating death, MI, or stroke, plus the occurrence of major bleeding, occurred in 12.7% of those using prasugrel and in 14.6% of the ticagrelor group (p=NS); specifically analyzing bleeding, it occurred in 8.1 and 10.6%, respectively (p=NS). Thus, in this subanalysis, both alternatives (prasugrel 5 mg or ticagrelor) demonstrated similar outcomes^13,14^.

### 4D acute coronary syndromes study^15^

In addition to P2Y12 inhibitor de-escalation, it also evaluated another strategy for prescribing the 5 mg/day dose of prasugrel: P2Y12 inhibitor monotherapy, designed to reduce bleeding events after PCI without interfering with major cardiovascular events. This clinical trial involved 656 patients with ACS undergoing PCI, who could be randomized to two strategies: (1) ASA+prasugrel 10 mg/day (or 5 mg/day for age >75 years or weight <60 kg) for 30 days, followed by monotherapy with prasugrel 5 mg/day for another 11 months; (2) ASA+prasugrel 5 mg/day for 12 months. The composite primary endpoint included death, MI, stroke, ischemia-driven revascularization, or bleeding at 1 year of follow-up. At the end of this period, a significant reduction in events was observed in the monotherapy group (4.9 vs. 8.8%; p=0.014). Major bleeding was observed more than seven times more frequently in the dual therapy group compared to the monotherapy group (0.6 vs. 4.6%; p=0.007). Thus, taking a 1-month prasugrel-based dual antiplatelet therapy followed by a lower, single dose of prasugrel reduced overall major adverse events by nearly half; this benefit came mainly from a 77% decrease in bleeding complications.

### Prasugrel in the 3.75 mg presentation

It is only available in the Asian continent, with mentions of its use in some publications from that region. Due to its unavailability in our environment and the absence of mention in the Guidelines, it will not be discussed in this review^2,4,16,17^.

### Executive summary

Table 1 outlines the main results of all the discussed trials.

**Table 1:** Main findings of the key studies analyzed (primary endpoint and major bleeding).

Events\Trial	Trilogy^9^ (7.243P)	Elderly ACS 2^11^ (1.443P)	Host-reduce^12^ (2.338P)	Isar react 5 substudy^14^ (1.099P)	4D ACS^15^ (656P)
Primary EP (%)	Pr 18.7×C 20.3 p=NS	Pr 17.0×C 16.6 p=NS	Pr 5 mg 7.2×Pr 10 mg 10.1 p<0.0001	Pr 12.7×T 14.6 p=NS	Pr 5 mg 4.9×Pr 10+5 mg 8.8 p=0.014
Bleeding (%)	Pr 2.5×C 1.8 p=NS	Pr 4.1×C 2.7 p=NS	Pr 5 mg 2.9×P 10 mg 5.9 p<0.0007	Pr 8.1×T 10.6 p=NS	Pr 5 mg 0.6×Pr 10+5 mg* 4.6 p=0.007

P: patients; EP: endpoint; Pr: prasugrel; C: clopidogrel; T: ticagrelor; ACS: acute coronary syndromes.

* Pr 10+5 mg: prasugrel 10 mg or 5 mg in those with age >75 years/weight <60 kg.

### Clinical implications - prasugrel 5 mg

- Pharmacodynamic studies have demonstrated the feasibility of using this presentation^4,7,8^.
- It is indicated in patients aged >75 years and weighing <60 kg. It aims to avoid the excess of bleeding complications in these populations while maintaining the positive impact on reducing major cardiovascular events^4,8,13,14^.
- Any presentation of prasugrel is formally recommended only in the ACS setting and in cases undergoing PCI, being contraindicated in those with a history of cerebral ischemia^2,4,13^.
- The Guidelines contemplate this possibility, although there are no conclusive clinical studies regarding the risk/benefit ratio compared to other alternatives^2,4,13–15^.

## CONCLUSION

A lower dose (5 mg once a day) of Prasugrel is indicated for patients with ACSs treated by PCI and at a higher risk of bleeding/ischemic events, more specifically those with a body weight of less than 60 kg or an age greater than 75 years.

## Data Availability

The datasets generated and/or analyzed during the current study are available from the corresponding author upon reasonable request.
